# Scientific Items

**Published:** 1886-10-20

**Authors:** 


### SCIENTIFIC ITEMS.

**Cause of the Charleston Earthquake.**—In your issue of the 18th September you publish a letter, over the signature Edward W. Byrn, which suggests a theory accounting for the Charleston earthquake.

Mr. Bryn takes the ground that "the escape of the vast volumes of petroleum and natural gas from the wells sunk into the bowels of the earth may furnish a cause for the earthquake in this region," and that these materials issuing from internal cavities release the superincumbent strata of rock, which, in consequence, falls, and the ensuing earth tremors are most severely felt along the line of weakness near the Atlantic coast.

In the first place, this theory takes for granted that either (1) these cavities have *alway* been full of natural gas and petroleum at a fixed pressure, or else (2) that in their formation and storing, the earth's crust was lifted and cavities made, which were immediately filled and supported the rock, as on a cushion.

Now:

1. Almost, if not all the theories accounting for the presence of gas and petroleum accept the fact that these substances were formed subsequent to the final comparative quiescence of the earth's crust after ages of upheavals and disturbances. This being so, there must have been cavities for the reception of gas and petroleum before they were formed; and, if the overlying strata kept their position, then, when the cavities were empty, why should they now be disturbed by the withdrawal of the contents of the cavities?

The "enormous pressure" at which they are sometimes found is easily accounted for by the continued accumulation of the gas and oil confined space, as the increase of pressure would by no means hinder the chemical combination of the materials of which they are formed.

2. That the pressure has raised the strata and made the cavaties is highly improbable, to say the least. That a pressure of some hundred pounds should raise a mass of rock from 1 to 2,000 feet in thickness and indefinite surface is beyond possibility.

As a matter of fact, no disturbance in the gas and oil regions was noticed at the time of the earthquake, and up to the present time no increase or diminution of the supply has been reported from any part of these districts.

I think Mr. Byrn's theory is entirely untenable, unless we radically change our views of the formation and storing of gas and petroleum.—*Scientific American.*

**Influence of Magnetism on Chemical Reaction.**—Mr. E. L. Nichols, in the *Journal of the Chemical Socioty*, describes a set of experiments with aquia regia, nitric acid, hydrochloric acid, and sulphuric acid to illustrate the phenomenon that when finely divided iron is placed in a magnetic field of considerable intensity and exposed to the action of the acid, the chemical reaction differs in several respects from that which occurs under ordinary circumstances. With aquia regia, it was found that the speed of reaction was greater in the magnetic field than without, and that the heat of chemical union is much greater. With nitric acid the effect of the magnet was to greatly increase the speed, reducing the average time from eight minutes to less than one minute. With sulphuric acid, the reaction was uniform and complete, and apparently of the same chemical character within and withoat the fluid. The magnet was found, however, to increase the speed of the reaction, and to decrease the amount of heat produced. A series of measurements was made with nitric acid, in which powdered copper was substituted for iron. The reaction in the field was found to be identical with that which occurred when the magnet was not in action.—*Ibid.*

**Machine to Make Conductors Honest.**—A checking apparatus for indicating and checking distances traveled by passengers on tramcars, omnibuses, cabs, and other vehicles is being made by Mr. H. Woolfe, of Barrington Road, Liverpool. The apparatus is small, and is to be fixed in a conspicuous position at the entrance of the car, and connected with the axle or wheel. The hand on a dial indicates the distance traveled. A gong on the top of the apparatus sounds every quarter of a mile, and the figures on the stamping or checking apparatus alter every quarter of a mile, correspounding with the number of miles indicated on the dial. The passenger on entering the car receives from the guard a ticket, which is stamped by the apparatus with the number of miles then shown on the indicator. This ticket is retained until the passenger gets off, when a glance at the indicator shows exactly how far he has traveled, and he pays accordingly. The guard again stamps the ticket, and the difference between the two numbers stamped thereon is the distance traveled, which must be accounted for by the guard when he delivers up his tickets at the office every journey.—*Scientific American.*

**Electrical Foot Warmers.**—The acetate of soda foot warmers now used for French railway carriages gradually become cool by radiation; but M. Tommasi, the French electrician, proposes to keep them up to a certain temperature by means of the heat due to an electric current traversing a high resistance. Only the heat lost by radiation is thus compensated for, so that the original high temperature is obtained on the cheaper plan of heating by fire, or rather by plunging the warmers in boiling water. The current employed to maintain their heat is to be supplied by a dynamo driven off an axle of the train, and the circuit passes through all the warmers. A simple device allows of the foot warmer being thrown unbearably hot. The plan will require fewer foot warmers than are now used, since it will be unnecessary to change them during a lengthy journey. This combination of fire and electricity heating is perhaps more likely to be successful for the present than a purely electrical arrangement. Warmers on the latter plan which have been devised are of feeble power.—*Ib.*

